# Molecular Identification of Insects on *Imperata cylindrica* (Poales: Poaceae) and Exploration of Potential Natural Enemies Using DNA Barcoding

**DOI:** 10.3390/insects17100990

**Published:** 2026-09-25

**Authors:** Dianyu Liu, Zhihan Su, Wenyan Xu, Yu Chen, Xiaoyu Yan, Yichen Wang, Chenxi Liu, Dean Brookes

**Affiliations:** 1 Sino-American Biological Control Laboratory, Institute of Plant Protection, Chinese Academy of Agricultural Sciences, No. 2 Yuanmingyuan West Road, Haidian District, Beijing 100193, China; liudianyu0808@163.com (D.L.); suzhihan0505@163.com (Z.S.); xuwenyan0222@163.com (W.X.); chenyu2632@163.com (Y.C.); yanxiaoyu0316@163.com (X.Y.); wangyichen2455@163.com (Y.W.); 2 USDA ARS Australian Biocontrol Laboratory, Ecosciences Precinct, 41 Boggo Road, Dutton Park, QLD 4102, Australia

**Keywords:** *Imperata cylindrica*, DNA barcoding, biological control, Chloropidae, *COI* gene, insect diversity

## Abstract

*Imperata cylindrica* is a highly invasive grass that causes severe ecological and agricultural damage worldwide, yet effective and sustainable management options remain limited. In this study, we investigated insects associated with the stems and rhizosphere soil of *I. cylindrica* in four parks in Beijing, China, and evaluated the utility of DNA barcoding for species identification and natural enemy discovery. A total of 16 insect samples were collected, and the mitochondrial *COI* gene was amplified using universal (LCO1490/HCO2198) and degenerate (KC-*COI*-F/R) primers. High-quality sequences were obtained from 12 of the 16 individual specimens, representing two classes (Insecta and Symphyla), two orders (Diptera and Coleoptera), and five families (Chloropidae, Muscidae, Rhiniidae, Eumolpidae, and Scutigerellidae). Chloropidae larvae (Diptera) dominated the stem-associated samples, and *Pseudocolaspis* sp. (Coleoptera: Eumolpidae), *Atherigona orientalis* (Diptera: Muscidae), *Rhyncomya pruinosa* (Diptera: Rhiniidae), and a species of Scutigerellidae (Symphyla) were also identified. BLAST analysis assigned the chloropid sequences to Chloropidae sp. BIOUG02404-H03 with 96.93–97.34% identity, and these sequences were recovered from stems at all four sampling sites. These results demonstrate that DNA barcoding effectively identifies morphologically challenging immature stages and soil-dwelling specimens. The abundant chloropid larvae, which feed internally in *I. cylindrica* stems, represent a promising candidate for further host-specificity testing as a classical biological control agent. This study provides the first molecular characterization of insects associated with *I. cylindrica* in Beijing and highlights the value of DNA barcoding in biocontrol resource discovery.

## Simple Summary

*Imperata cylindrica* is one of the most damaging invasive weeds worldwide, yet sustainable control options remain limited. This study aimed to identify insects associated with *I. cylindrica* in Beijing and to evaluate their potential as biological control agents. Insects were collected from stems and rhizosphere soil in four parks, and DNA barcoding was used to identify morphologically challenging larvae and pupae. From 16 samples, 12 yielded high-quality *COI* sequences representing two classes, two orders, and five families. Chloropidae larvae dominated the stem-associated samples and may have potential as biological control agents. These results show that DNA barcoding is effective for identifying immature and soil-dwelling specimens, and provide baseline data for natural enemy discovery against this invasive weed.

## 1. Introduction

*Imperata cylindrica* (L.) Beauv. (Poales: Poaceae) is a perennial grass native to Asia and widely recognized as one of the most damaging invasive weeds globally. It ranks as the seventh most problematic perennial plant worldwide, negatively affecting 35 crops across 73 tropical regions and causing substantial economic and environmental damage [1,2,3]. The species is broadly distributed across tropical, subtropical, and temperate zones, exhibiting high ecological adaptability and competitive ability. Through clonal propagation via an extensive rhizome system and prolific seed production, *I. cylindrica* rapidly forms monodominant stands in various habitats [4,5,6]. Studies have shown significant competitive effects on native plants, leading to reduced biodiversity and impaired ecosystem functions [2]. In the southeastern United States, its invasion poses serious threats to agriculture, forest regeneration, and ecological security [2,7,8].

*I. cylindrica* is a perennial rhizomatous grass. It produces extensive, deeply penetrating rhizomes and dense fibrous roots, and its stems are erect, slender, and often form dense monodominant stands. The rhizome system enables rapid clonal spread and makes mechanical control difficult because underground fragments can regenerate. The stem structure and rhizosphere environment provide concealed microhabitats for stem-boring and soil-dwelling arthropods, which are the focus of this study.

Current management relies primarily on chemical herbicides and mechanical tillage, both with limited efficacy. Chemical control is costly and poses environmental risks, including off-target toxicity; mechanical control often fails to remove underground rhizomes completely, leading to regrowth [9]. Therefore, developing safe and sustainable control strategies is urgently needed.

Biological control is considered one of the most promising sustainable approaches for managing invasive plants. The fundamental strategy involves introducing specialized natural enemies (insects, pathogens) from the native range of the invasive species to suppress its growth and reproduction, ultimately reducing it below economic or ecological thresholds [10]. The introduction and successful establishment of the gall fly *Procecidochares utilis* Stone, 1947 (Diptera: Tephritidae) against *Ageratina adenophora* (Spreng.) R.M. King & H. Rob., 1970 (Asterales: Asteraceae) is a classic example [11]. For *I. cylindrica*, several biocontrol efforts have been undertaken internationally. Early research focused on fungal bioherbicides; for instance, *Bipolaris sacchari* and *Drechslera gigantea* isolated from diseased *I. cylindrica* in Florida caused severe leaf spotting and blight in greenhouse and field trials, showing potential as mycoherbicides [12]. Concurrently, surveys for insect natural enemies have progressed. In a joint US–Japan survey, the stem-boring noctuid *Acrapex azumai* Sugi, 1970 (Lepidoptera: Noctuidae) was found feeding at stem bases of *I. cylindrica* in Kyushu, Japan, causing “dead heart” symptoms. Its identity was confirmed via morphology and *COI* barcoding, marking the first host record and suggesting its potential as a specialist natural enemy [13]. Subsequent field surveys in Tanzania, Uganda, the Philippines, and Indonesia identified additional candidates, including *Acrapex* spp., *Emmalocera* Ragonot, 1888 (Lepidoptera: Pyralidae) and *Chilo* Zincken, 1817 (Lepidoptera: Crambidae) larvae, and the stem-galling midge *Orseolia javanica*, all exhibiting strong host specificity, providing new resources for classical biocontrol of *I. cylindrica* [14]. Stem-boring insects, due to their concealed life history and often narrow host ranges, are prime targets in weed biocontrol programs. Additionally, soil insects, as key components of belowground ecosystems, interact with plant roots and may also influence *I. cylindrica* growth.

Traditional morphological identification of insects faces significant limitations, particularly for larvae, females of some species, and cryptic or closely related taxa [15,16,17]. Many immature stages lack diagnostic morphological characters, and small soil-dwelling specimens are frequently damaged, making accurate identification challenging. DNA barcoding has been widely used to identify such specimens [18,19,20]. The mitochondrial cytochrome c oxidase subunit I (*COI*) gene, as the standard DNA barcode marker, has been widely adopted for insect species identification [18]. This technique amplifies a standard fragment (typically ~650 bp) and compares the obtained sequence against reference databases (GenBank, BOLD), enabling rapid and accurate species identification. Studies have demonstrated high effectiveness of DNA barcoding for both identifying known species and discovering previously unrecognized species [21,22].

In biocontrol agent screening, DNA barcoding not only accurately identifies target insects but also provides essential data for subsequent population genetic structure and host-specificity studies. Combining morphological and molecular identification has become the standard approach for insect diversity surveys and natural enemy discovery.

This study targeted *I. cylindrica* plants with chlorotic, wilted, or dead central stems in four parks in Beijing, based on the hypothesis that such symptoms may indicate the presence of stem-feeding insects. Insects were collected from stems and rhizosphere soil. Larvae and pupae were first examined morphologically; when morphological characters were insufficient for reliable identification, DNA barcoding was used for molecular identification. The objectives were: (1) to identify insects associated with *I. cylindrica* stems and rhizosphere soil in Beijing; (2) to screen for taxa with potential as biological control agents; and (3) to assess the utility of combining larval/pupal morphology with DNA barcoding for identifying morphologically difficult specimens. The findings will provide baseline data for biocontrol resource exploration against *I. cylindrica* and inform molecular identification methods for insect diversity associated with invasive plants.

## 2. Materials and Methods

### 2.1. Study Area and Sample Collection

The study was conducted in Beijing, China, at four sampling sites: Nanhaizi Park (Nh), Olympic Forest Park North (On), Olympic Forest Park South (Os), and Kangxi Wetland Forest Park (Kx) (Figure 1). Field surveys were conducted during the peak growing season of *I. cylindrica* in September 2025. In *I. cylindrica*, the culm is enclosed by leaf sheaths at the base, and the rhizome system extends belowground. During field surveys in the four parks, some *I. cylindrica* plants showed wilting and yellowing of the central stem, suggesting possible insect feeding inside the stem. These plants were collected and dissected in the laboratory, and larvae and pupae were found inside the stems. Stems and rhizosphere soil were therefore sampled to target stem-inhabiting and soil-dwelling insects. Each target plant was carefully excavated, including aboveground stems and the surrounding rhizosphere soil (5 cm diameter, 15 cm depth). Samples were preliminarily examined in the field, then placed into sealed plastic bags and transported to the laboratory.

In the laboratory, each sample was dissected manually. Stems were split open and examined, and rhizosphere soil was placed into white porcelain trays for careful sorting of motile insects using forceps and dissecting needles. All collected insects (larvae, pupae, and adults) were immediately transferred to 15 mL screw-cap tubes containing 95% ethanol for preservation. Each tube was labeled with a unique code (e.g., Nh-1). A total of 16 insect-containing samples were obtained: five from Nanhaizi Park, four from Olympic Forest Park North, three from Olympic Forest Park South, and four from Kangxi Wetland Forest Park. All specimens were preserved in 95% ethanol at 4 °C in the dark until DNA extraction. DNA was extracted within 2 weeks after collection.

All specimens were first examined morphologically under a stereomicroscope. Larvae and pupae were classified into morphotypes based on body shape, coloration, segmentation, and presence or absence of appendages. When morphological characters were insufficient for reliable identification, specimens were subjected to DNA barcoding. This study focused on stem-inhabiting and soil-dwelling insects, particularly larvae and pupae. Mites and other microarthropods were not surveyed.

### 2.2. DNA Extraction, PCR Amplification, and Sequencing

Each specimen was labelled individually in the field and processed separately in the laboratory. Total DNA was extracted from each specimen, and the COI gene was amplified with standard barcoding primers. Amplification success was verified by agarose gel electrophoresis, and the PCR products were sequenced bidirectionally. The resulting sequences were compared against the NCBI database using BLAST.

Each insect specimen was removed from 95% ethanol and rinsed three times with sterile water to remove residual ethanol. Total genomic DNA was extracted using the TIANamp Micro DNA Kit (Tiangen Biotech, Beijing, China) following the manufacturer’s instructions.

The universal primers LCO1490/HCO2198 are standard *COI* barcoding primers for metazoan invertebrates. First-round PCR amplification was performed using universal primers LCO1490 (5′-GGTCAACAAATCATAAAGATATTGG-3′) and HCO2198 (5′-TAAACTTCAGGGTGACCAAAAAATCA-3′) [23], targeting an ~650 bp fragment of *COI*. The 25 µL reaction mixture contained: 2.0 µL DNA template, 1.25 µL each of forward and reverse primers (10 µM), 12.5 µL Q5 High-Fidelity 2X Master Mix (New England Biolabs, Ipswich, MA, USA), and 8.0 µL nuclease-free water. PCR conditions were: initial denaturation at 98 °C for 30 s; 34 cycles of 98 °C for 10 s, 58 °C for 30 s, 72 °C for 45 s; and final extension at 72 °C for 5 min. The degenerate primers KC-COI-F/R contain degenerate bases and were used as a supplement for samples that failed to amplify or produced double peaks with the universal primers. For samples that showed double peaks or failed amplification in the first round, a second round was performed using degenerate primers KC-*COI*-F (5′-TYTCAACAAAYCYAARGATATTGG-3′) and KC-*COI*-R (5′-TAAACTTCWGGRTGWCCAAARAATCA-3′) [24] under identical reaction conditions. PCR products were visualized on 1% agarose gels (Invitrogen, Waltham, MA, USA). Positive products were purified using the AxyPrep DNA Gel Extraction Kit (Axygen, Union City, CA, USA) and sequenced bidirectionally on an ABI 3730XL sequencer (Applied Biosystems, Waltham, MA, USA).

### 2.3. Sequence Processing and Species Identification

Sequence chromatograms were inspected using Chromas 2.4.1, and sequences were assembled and trimmed using ContigExpress (20 June 2000) SeqMan software, removing low-quality bases and primer regions. The resulting *COI* sequences (length 450–600 bp) were compared against the GenBank database using NCBI BLASTn (https://blast.ncbi.nlm.nih.gov/Blast.cgi, accessed on 26 March 2026). Species identification followed established criteria: sequences with ≥98% identity and ≥90% query coverage were assigned to species level; sequences with 95–98% identity were assigned to genus level; sequences with <95% identity were reported only at higher taxonomic levels (e.g., family or order). These thresholds follow commonly used operational criteria for *COI* barcoding [18,19,23]. Low-identity matches were treated as tentative identifications. Samples that yielded double peaks in sequencing or showed low BLAST confidence (identity < 95% or coverage < 90%) were recorded as tentative identifications and were not used for further ecological inference. Taxonomic classification followed the NCBI Taxonomy database, using standard Linnaean ranks down to the family level.

## 3. Results

### 3.1. Sampling Overview

A total of 16 insect samples were collected from stems and rhizosphere soil of *I. cylindrica* at four Beijing sites. Preliminary morphological examination revealed that most samples were dipteran larvae or pupae lacking stable diagnostic characters, precluding accurate morphological identification (Figure 2, Figure 3, Figure 4 and Figure 5). The specimens were assigned to larval, early pupal, and pupal stages based on visible features. On-1 and On-3 were larvae; On-1 had a milky white to pale yellow body with visible mouthparts, legs, and numerous long setae; and On-3 had a light brown, smooth, cylindrical body with clearly visible segments, mouthparts, and legs. On-2 was at the early pupal stage, with a pale yellow body, setae, and larval head features, but larval legs were not visible. Kx-1, Kx-2, Kx-3, and Kx-5 were early pupae with pale yellow to milky white bodies and visible segments. Kx-4, Nh-1 to Nh-4, On-4, Os-1, Os-2, and Os-3 were pupae with amber to dark brown bodies and visible segments. On-4 showed clearly visible segmentation, with approximately eight body segments countable. Larval mouthparts and legs were not visible in any pupal specimen. Body color was recorded for all specimens.

When using universal primers LCO1490/HCO2198, 7 of 16 samples showed double peaks in sequencing (Nh-1, Nh-2, Os-2, On-2, Kx-3, Kx-4, Kx-5), and one sample (Nh-3) had low BLAST identity (92.39%). For samples with failed or unreliable first-round results, degenerate primers KC-*COI*-F/R were used. Ultimately, high-quality *COI* sequences (450–600 bp, mean GC content 35.2%) were obtained from 12 of the 16 samples (Figure 6). Four samples (Nh-1, Os-2, Kx-4, Kx-5) showed double peaks or no amplification even after the second round and were recorded as identification failures.

### 3.2. Species Identification Results

BLAST analysis against GenBank successfully identified the 12 samples. Taxonomic classification followed the NCBI Taxonomy database down to the family level (Table 1). The 12 samples belonged to two classes (Insecta and Symphyla), two orders (Diptera and Coleoptera), and five families (Chloropidae, Muscidae, Rhiniidae, Eumolpidae, and Scutigerellidae). Diptera was the most abundant order, including: an unidentified chloropid species Chloropidae sp. BIOUG02404-H03 (samples Nh-4, Os-1, Os-3, Kx-1, Kx-2; 96.93–97.34% identity); another chloropid *Tricimba* sp. TAW1 (Nh-2; 91.98% identity, low coverage, identified to genus level); *Atherigona orientalis* (On-4; 99.55% identity); and *Rhyncomya pruinosa* (Nh-3; 92.39% identity, tentative; Diptera: Rhiniidae). Coleoptera included two species: *Pseudocolaspis* sp. EMHAU-15070625 (On-1, On-3; 99.11–99.26% identity) and *Athous* sp. BBCCN394-10 (On-2; 85.4% identity, very low coverage, tentative). Additionally, a Symphylan species of Scutigerellidae (Kx-3; 98.64% identity) was identified from soil samples at Kangxi Forest Park.

Overall, chloropid larvae dominated the stem-associated insects (5 successfully identified samples), while soil insects included coleopteran larvae/adults and a symphylan.

### 3.3. Screening of Potential Natural Enemy Taxa

Based on feeding habits and ecological functions, the following taxa were preliminarily identified as having potential biocontrol value: (1) Chloropidae larvae, which bore inside *I. cylindrica* stems, potentially causing mechanical damage and disruption of nutrient transport—their host specificity warrants further evaluation; (2) *Atherigona orientalis*, whose larvae are often found in decaying plant material—whether it actively attacks healthy *I. cylindrica* remains unclear; and (3) soil symphylan Scutigerellidae—although not a direct natural enemy, its rhizosphere activity might indirectly affect root health.

## 4. Discussion

In this study, *I. cylindrica* plants with chlorotic, wilted, or dead central stems were sampled based on the hypothesis that stem-feeding insects may be associated with these symptoms. DNA barcoding identified 12 of 16 insect samples. The dominant taxon was Chloropidae sp. BIOUG02404-H03, which was recovered from stems at all four sampling sites. This is the first molecular record of chloropid larvae associated with *I. cylindrica* in northern China.

Chloropid larvae are often stem-boring or leaf-mining in grasses, and some species have been considered as potential biocontrol agents of invasive grasses [25,26]. For example, *Platylephala planifrons* has been reported to reduce aboveground biomass of *Phragmites australis* by boring into stem bases [27]. The feeding ecology and host range of the chloropid species detected here remain unknown. Its occurrence in *I. cylindrica* stems at all four sites makes it a candidate for host-specificity testing.

To further corroborate the molecular identification of this morphologically challenging taxon, the *COI* sequences matching Chloropidae sp. BIOUG02404-H03 were queried against the BOLD database. This search resulted in a match to the Barcode Index Number BIN BOLD: ABX2831 (Figure 7A,B). Although direct morphological comparison with our immature specimens was not feasible, the reference images of adult flies associated with this BIN, recorded in Pakistan and Bangladesh, provide morphological context for the adult stage of this taxon. The geographic records of this BIN also indicate that this chloropid lineage occurs in South Asia, which is relevant to its potential host range and regional distribution.

*Atherigona orientalis* was identified from one sample with 99.55% identity. This species is a cosmopolitan saprophagous and facultatively phytophagous fly, and its polyphagous habits make it unlikely to be suitable as a classical biocontrol agent for *I. cylindrica* [28]. *Pseudocolaspis* sp. (Coleoptera: Eumolpidae) was found in rhizosphere soil at Olympic Forest Park North; although some chrysomelids are used in weed biocontrol [29,30,31], the host range of this species is unknown. The symphylid Scutigerellidae sp. is a soil-dwelling detritivore/fungivore [32] and is not expected to directly suppress *I. cylindrica*.

DNA barcoding identifications depend on the accuracy of reference sequences deposited in GenBank and BOLD. Public databases are known to contain misidentified or incomplete records; therefore, the species-level assignments in this study are provisional. The five chloropid sequences were identical or nearly identical to each other and matched the same reference sequence, which supports their assignment to Chloropidae. However, species-level identity requires corroboration by adult morphology or additional molecular markers. No adult rearing was performed in this study, and morphological verification was based on larvae and pupae only.

The present dataset consists of 16 samples collected during a single month (September 2025). Some BLAST matches had low identity (e.g., *Rhyncomya pruinosa* 92.39%, *Athous* sp. 85.4%), and these identifications are treated as tentative. Mites and other microarthropods were not surveyed. The four samples that failed to amplify may reflect DNA degradation during storage. No adult rearing was performed in this study, and morphological verification was based on larvae and pupae only.

Future work will expand sampling across seasons and additional regions to obtain a more complete picture of the arthropod community associated with *I. cylindrica*. Adults will be reared from immature specimens to allow morphological verification and more reliable species-level identification. Additional molecular markers will be used to corroborate the provisional species-level assignments. Host-specificity tests will be conducted for the chloropid candidate, and the potential role of soil-dwelling taxa in the rhizosphere will also be examined.

## 5. Conclusions

DNA barcoding identified insects associated with *I. cylindrica* in Beijing, yielding high-quality COI sequences from 12 of 16 individual samples representing two classes (Insecta and Symphyla), two orders (Diptera and Coleoptera), and five families (Chloropidae, Muscidae, Rhiniidae, Eumolpidae, and Scutigerellidae). Chloropidae species BIOUG02404-H03 dominated the stem-associated samples at all four sites and was assigned to BIN BOLD: ABX2831; it is the main candidate for further host-specificity testing as a classical biological control agent. Other taxa, including *Pseudocolaspis* sp., *Atherigona orientalis*, *Rhyncomya pruinosa*, and a Scutigerellidae species, were recorded but were not considered primary biocontrol candidates. This study provides the first molecular characterization of insects associated with *I. cylindrica* in northern China, with species-level assignments treated as provisional pending adult morphology or additional molecular markers.

## Figures and Tables

**Figure 1:**
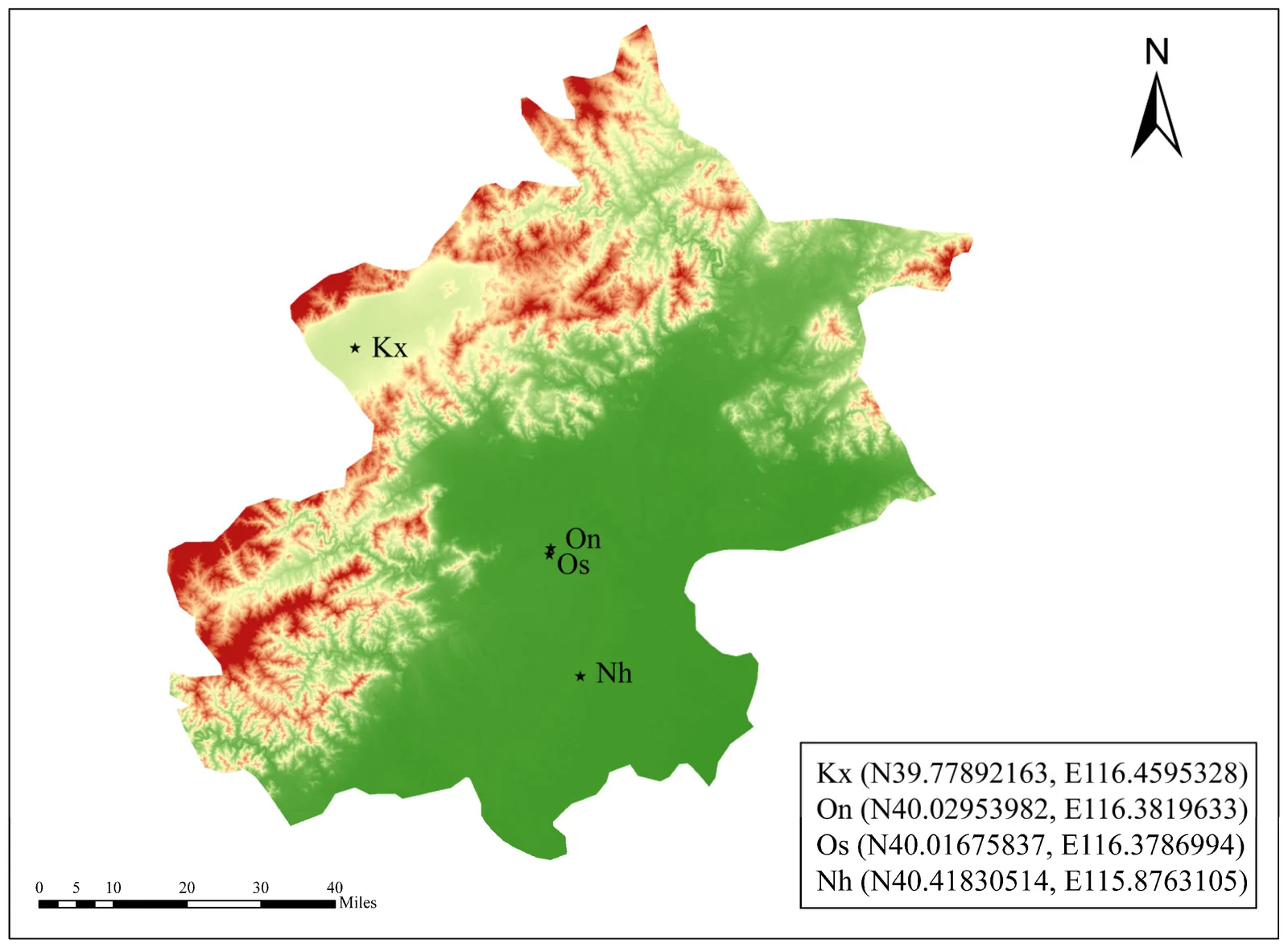
Caption: Map of sampling sites in Beijing parks. Nh: Nanhaizi Park; On: Olympic Forest Park North; Os: Olympic Forest Park South; Kx: Kangxi Wetland Forest Park. The stars represent the sampling sites in different parks, and the box provides the latitude and longitude coordinates for each sampling site.

**Figure 2:**
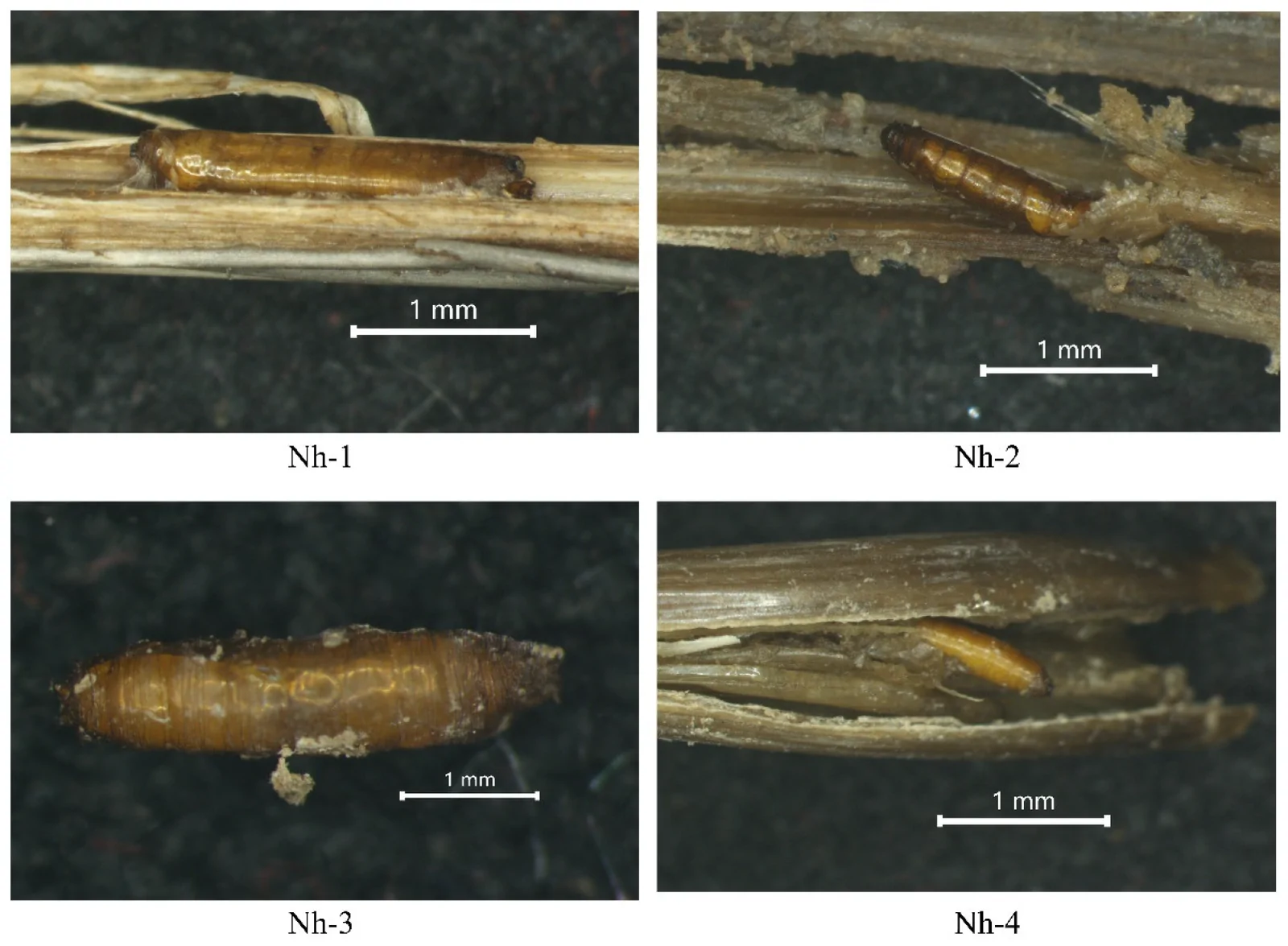
Morphological photographs of insect specimens collected from *I. cylindrica* at Nanhaizi Park (Nh). Samples Nh-1 to Nh-4. Scale bars = 1 mm.

**Figure 3:**
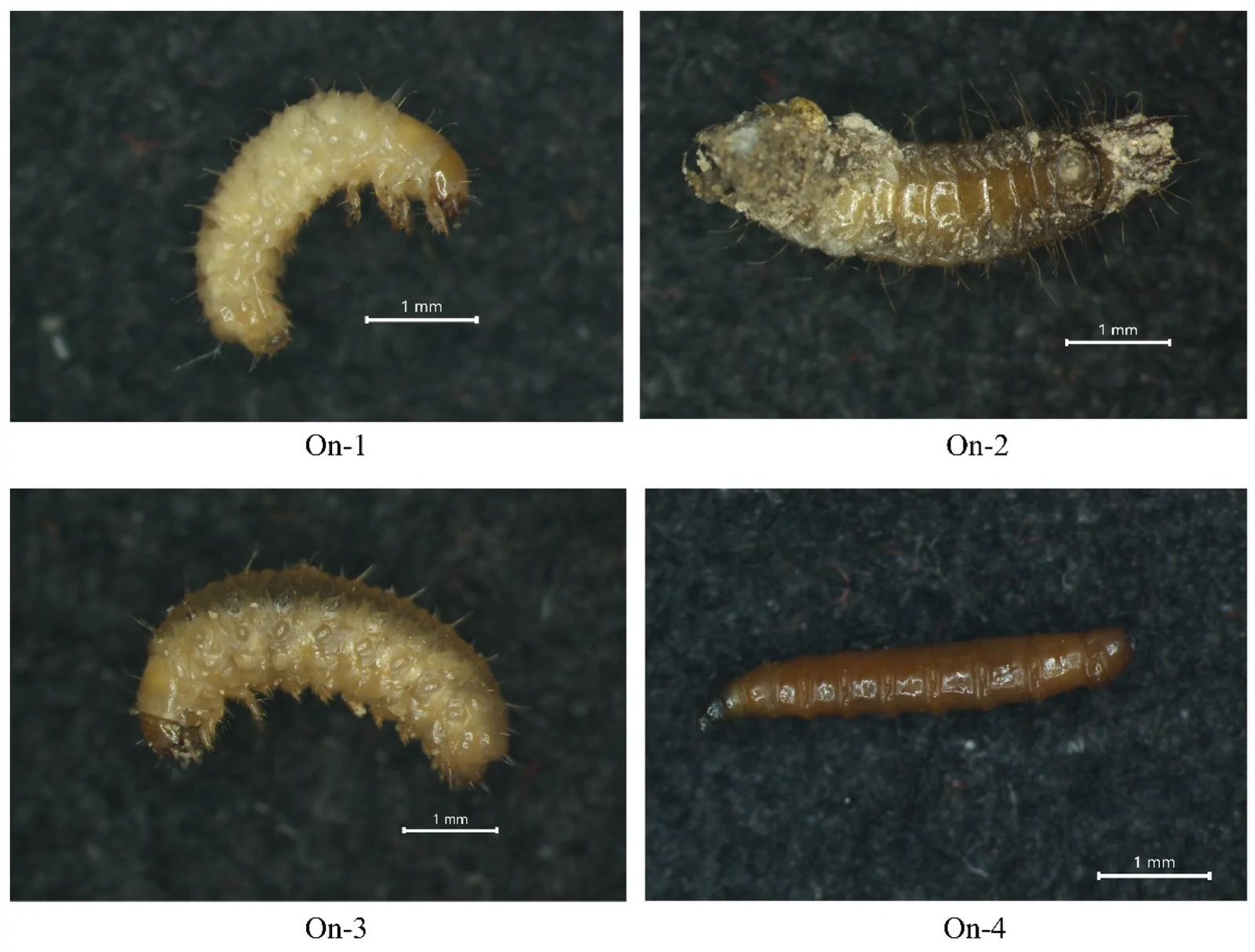
Morphological photographs of insect specimens collected from *I. cylindrica* at Olympic Forest Park North (On). Samples On-1 to On-4. Scale bars = 1 mm.

**Figure 4:**
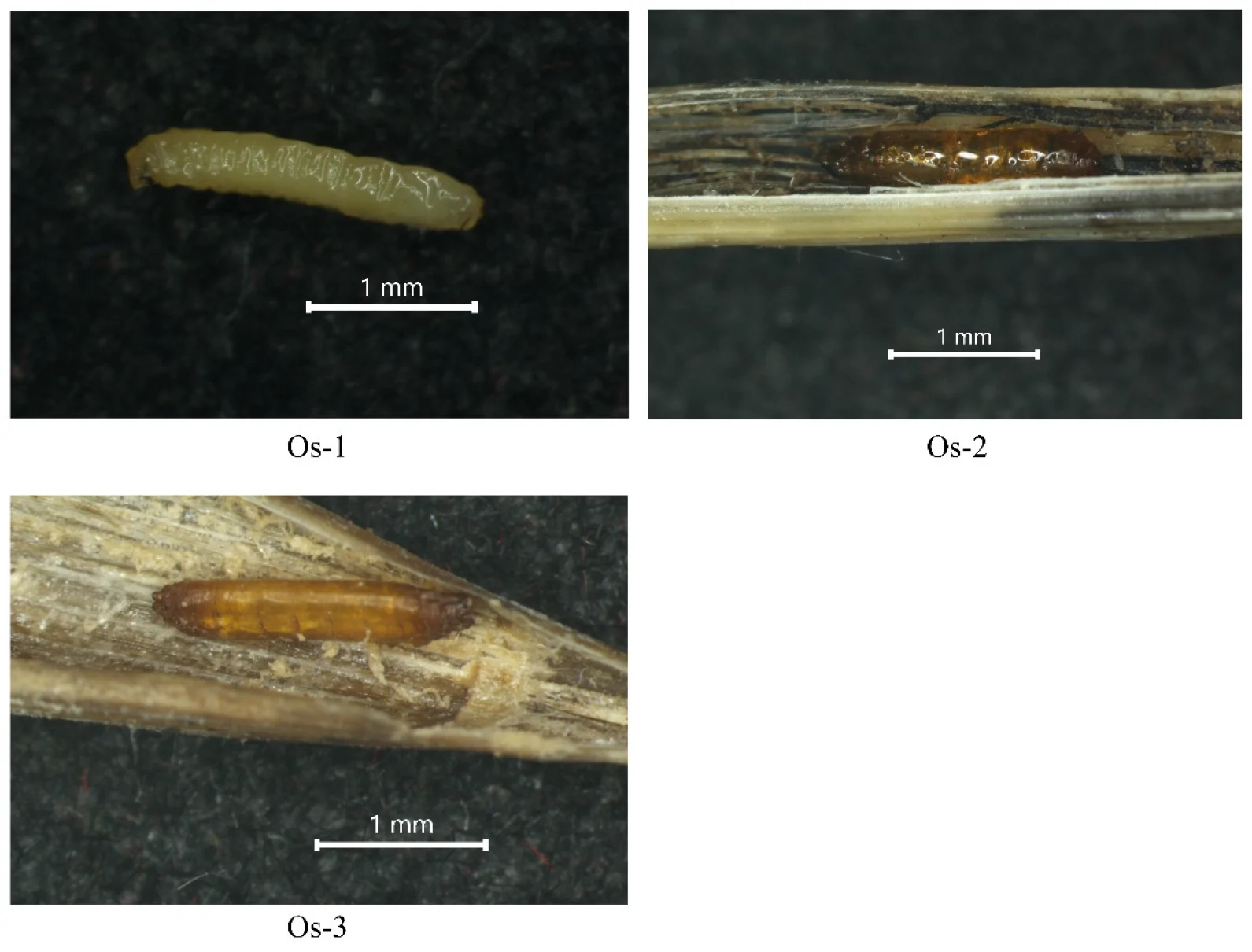
Morphological photographs of insect specimens collected from *I. cylindrica* at Olympic Forest Park South (Os). Samples Os-1 to Os-3. Scale bars = 1 mm.

**Figure 5:**
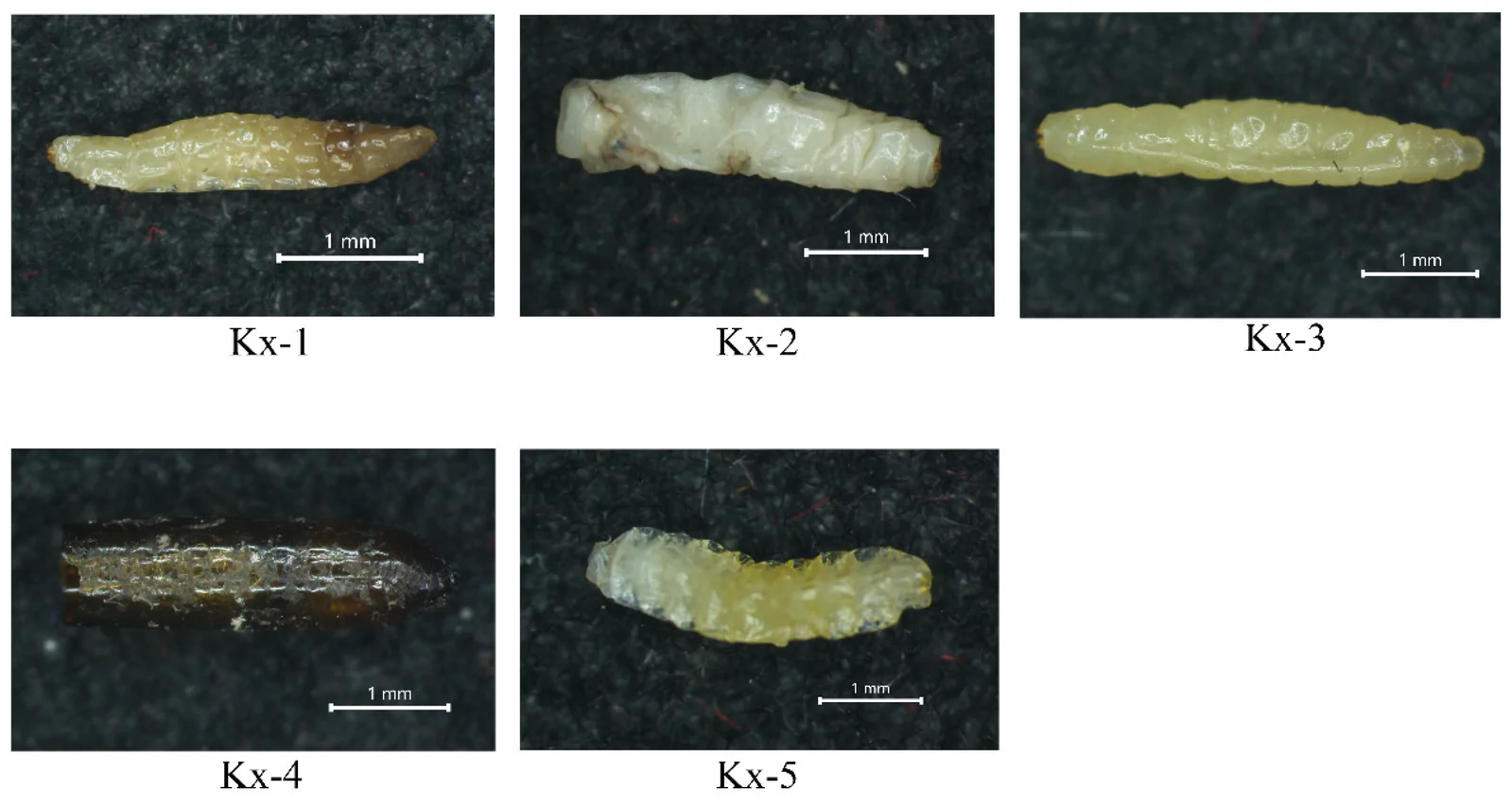
Morphological photographs of insect specimens collected from *I. cylindrica* at Kangxi Wetland Forest Park (Kx). Samples Kx-1 to Kx-5. Scale bars = 1 mm.

**Figure 6:**
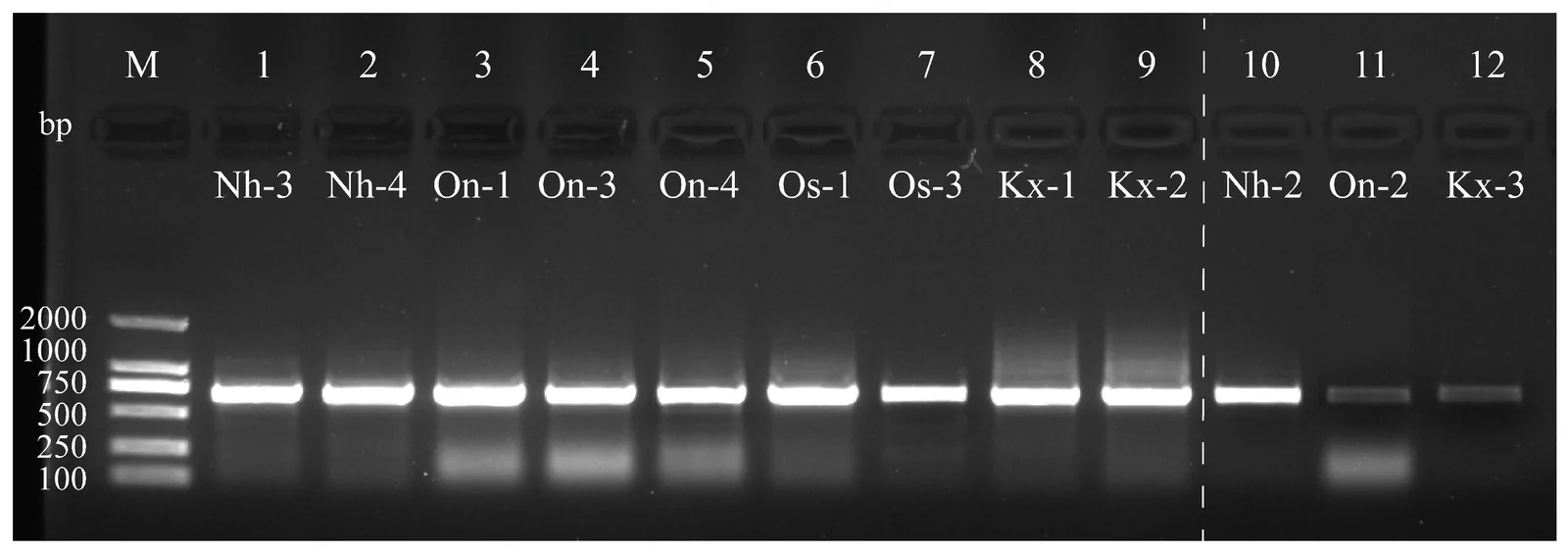
Agarose gel electrophoresis of *COI* gene PCR amplicons. Lane M: DNA marker (DL2000). Lanes 1–9: Samples amplified with universal primers LCO1490/HCO2198 (Nh-3, Nh-4, On-1, On-3, On-4, Os-1, Os-3, Kx-1, Kx-2), all showing specific bands at approximately 450–600 bp. Lanes 10–12: Samples amplified with degenerate primers KC-*COI*-F/KC-*COI*-R (Nh-2, On-2, Kx-3), all showing visible amplification bands at approximately 450–600 bp. The dash line separates the two groups of samples amplified with different primers.

**Figure 7:**
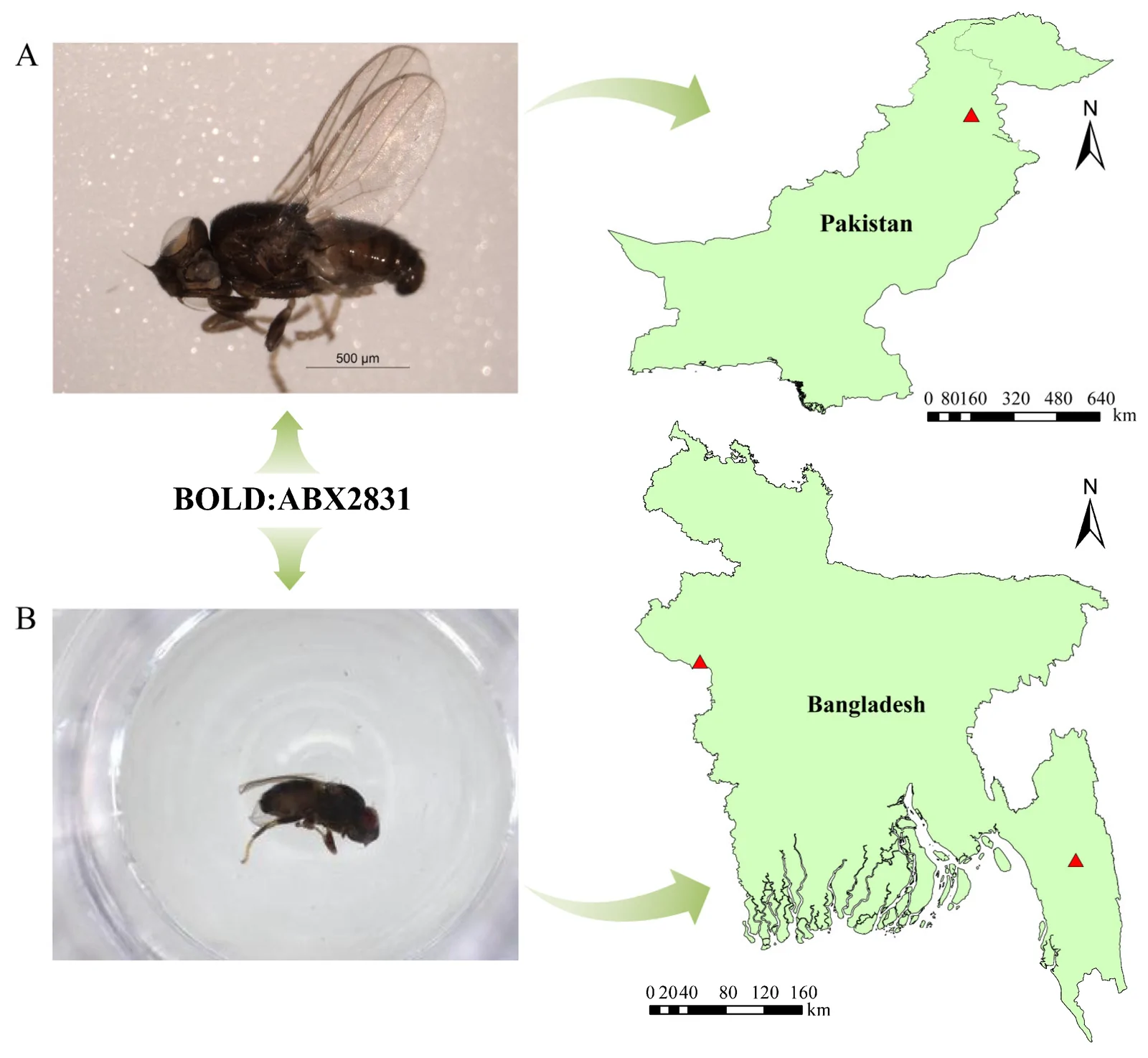
Molecular association and reference distribution data for the identified chloropid species (BIN: BOLD: ABX2831) retrieved from the BOLD database. (**A**) Lateral habitus of an adult specimen and its distribution map in Pakistan. The red triangle indicates the geographic origin based on BOLD records. (**B**) Lateral habitus of an adult specimen and its distribution map in Bangladesh. The red triangle indicates the geographic origin based on BOLD records. The central text “BOLD: ABX2831” and arrows link the specimens in (**A**,**B**) to the same Barcode Index Number (BIN), confirming their molecular association; the green arrows further indicate the geographic origin of the insect specimens shown in (**A**,**B**). Distribution maps were redrawn based on the coordinates provided in BOLD records. Image credits: BOLD (https://portal.boldsystems.org/bin/BOLD:ABX2831, accessed on 25 August 2026).

**Table 1:** Identification results using universal primers (LCO1490/HCO2198).

Primers	Sample ID	BLAST Top Hit	Taxonomic Group	Percent Identity (%)
LCO1490/HCO2198	Nh-3	*Rhyncomya pruinosa*	Diptera: Rhiniidae	92.39
	Nh-4	Chloropidae sp. BIOUG02404-H03	Diptera: Chloropidae	97.34
	On-1	*Pseudocolaspis* sp. EMHAU-15070625	Coleoptera: Eumolpidae	99.26
	On-3	*Pseudocolaspis* sp. EMHAU-15070625	Coleoptera: Eumolpidae	99.11
	On-4	*Atherigona orientalis*	Diptera: Muscidae	99.55
	Os-1	Chloropidae sp. BIOUG02404-H03	Diptera: Chloropidae	96.93
	Os-3	Chloropidae sp. BIOUG02404-H03	Diptera: Chloropidae	97.18
	Kx-1	Chloropidae sp. BIOUG02404-H03	Diptera: Chloropidae	97.34
	Kx-2	Chloropidae sp. BIOUG02404-H03	Diptera: Chloropidae	97.34
KC-*COI*-F/R	Nh-2	*Tricimba* sp. TAW1	Diptera: Chloropidae	91.98
	On-2	*Athous* sp. BBCCN394-10	Coleoptera: Elateridae	85.4
	Kx-3	Scutigerellidae sp.	Symphyla: Scutigerellidae	98.64

## Data Availability

Data will be made available upon reasonable request from the corresponding author.

## Abbreviations

The following abbreviations are used in this manuscript:

NCBI	National Center for Biotechnology Information
*COI*	cytochrome c oxidase subunit I
